# Special Issue “Mediterranean Diet and Metabolic Diseases”

**DOI:** 10.3390/nu13082680

**Published:** 2021-08-01

**Authors:** Emmanuella Magriplis, Michail Chourdakis

**Affiliations:** 1 Department of Food Science and Human Nutrition, Agricultural University of Athens, Iera Odos 75, 11855 Athens, Greece; 2 Laboratory of Hygiene, Social and Preventive Medicine and Medical Statistics, School of Medicine, Faculty of Health Sciences, Aristotle University of Thessaloniki, 54124 Thessaloniki, Greece

The Mediterranean diet (MD) has been considered among the healthiest dietary patterns since a little over 50 years ago, Ancel Keys—as the key figure—provided evidence for the beneficial effects of the MD. Furthermore, MD is recognized by UNESCO as an “Intangible Cultural Heritage of Humanity” since it is an integral part of tradition and heritage as well as one of the healthiest and most sustainable dietary patterns due to the fact that its baseline is plant-based foods, non-refined grains and cereals, and of course extra virgin olive oil. The MD has been inversely associated with various chronic and metabolic diseases, including cardiovascular disease and obesity, as well as with further metabolic factors. However, in past decades studies have shown that countries in the Mediterranean basin are drifting away from this prudent pattern and are adopting a more Westernized diet. This drift is due to many multidimensional and region-specific influences, including socioeconomic, sociodemographic, environmental and cultural/ethnic, as well as factors that interfere with food preferences/dislikes, with accessibility to foods and with religious beliefs. 

If present trends persist, it is expected that non-communicable diseases (NCD) will have a growing incidence, making the studies included in the present Special Issue (SI) imperative, since despite the first ecological and population-based data, the aspects of the MD that may promote health in respect to other lifestyle variables remain under investigation, as Balakoudi and colleagues concluded following a systematic review [1]. This SI contains multifaceted research articles and a systematic review, which have addressed these issues both in healthy individuals and in those with specifically defined disease states or risk factors. Specifically, this SI contains research that has been carried out in not only the general population, but also among patients with Type 1 diabetes mellitus (T1DM) [2] or dyslipidemia [3,4,5], as well as among firefighters who are proxy to healthy and physically fit individuals [6,7], adding valuable information to the pre-stated gap in knowledge.

A very interesting paper that has been included in the present SI examines MD adherence during the COVID-19 pandemic by Polish women diagnosed with T1DM2. The authors performed an online case control study during the peak of the second COVID-19 pandemic wave and used the Mediterranean Diet Adherence Screener (MEDAS) to assess the level of adherence to the MD. Overall, the authors found that the majority of participants had a moderate adherence and found that significantly more women with T1DM compared to healthy women adhered to olive oil, fruit and fish and seafood intake. However, a significantly larger percentage also exceeded butter consumption per day and did not adhere to red meat recommendations, indicating a higher saturated fat intake, a factor in contrast to the American Diabetes Association guidelines [8,9]. This is of importance considering the adverse effects of the COVID-19 virus on health, especially on individuals who are already at high risk, such as individuals with diabetes mellitus.

In another study, Formisano and her colleagues helped to address gaps among individuals with dyslipidemia. another risk factor for metabolic diseases, especially atherosclerotic cardiovascular disease. They aimed to evaluate how adherence to the MD affects the lipid profile in patients with dyslipidemia, using a retrospective design [3]. Elevated levels of low-density lipoprotein cholesterol (LDL-c) and triglycerides (TG), with low levels of high-density lipoprotein cholesterol (HDL-c), represent the most atherogenic dyslipidemic profile, which has been shown to be potentially ameliorated from a higher MD adherence. The authors reported the beneficial effects of a higher MD adherence on both HDL-c and TG levels, the latter coinciding with more frequent olive oil intake, the main denominator of the MD. Results on HDL-c were in agreement with a cross-sectional study [7] and an intervention study [6] on healthy volunteers also included in the issue [6], and results on HDL-c and TG are in agreement with the systematic review published on metabolic syndrome specific risk factors [1]. The authors found that nutritional counselling improved adherence, as was also suggested by Grabia and colleagues in their study on T1DM women [2]. The authors also addressed the main conflicting issue today with regard to recommended intakes of total saturated fats, and based on their findings they suggested that foods containing saturated fats may need to be distinguished when researched. This suggestion pertains to investigating the whole food instead of specific macronutrients, in order to consider interactions, instead of the nutrient alone. 

Four of the studies included in this issue have been carried out in healthy volunteers [4,5,6,7]. Romanidou and colleagues reported that for a unitary increase in MD adherence, the ratio of total cholesterol to HDL-c decreased by 0.03 and HDL-c increased by 0.25 mg/dL in 460 firefighters, after adjusting for multiple confounders [7]. Two of the studies examined adults residing in the United Arab Emirates (UAE), one of the countries that makes up the Gulf Cooperation Council that has seen a rapid improvement in socio-economic status and a simultaneous dietary transition. This dietary transition, characterized by an increase in processed food and meat intake—both regarded as factors that act as a mediator to overweight and obesity, metabolic syndrome and other risk factors—has contributed to the estimated high mortality from NCD’s in these regions in total (65–68%); in UAE, CVD accounts for 25% of all deaths, a value that reaches 29% in Abu Dhabi. 

Jarrar and his colleagues evaluated urinary sodium excretion using 24-h urine samples, the ideal methodology for evaluating sodium intake from food and added salt during cooking or at the table [4]. Sodium is the mineral that has received great attention in past years, and it is widely accepted that intakes above 2300 mg increase the risk of hypertension. Sodium, other than salt, is found in high concentrations in processed foods and meat—foods that characterize Western-type diets—whereas it is low in fruit, vegetables, and legumes—foods that prevail in the MD. The authors reported that 67.4% of the population that was assessed exceeded the recommended intake of 5–6 g of salt per day, with an average of 10.4 g per day, although only 45.5% reported always adding salt to their food and only 20% were aware of their overconsumption. 

These results in combination with the unhealthy dietary patterns reported by Ismail and colleagues in UAE adults [5] reveal the extent of the problem in this region. The authors performed an online study to assess dietary habits and other behaviors during the COVID-19 lockdown. This was imperative since such measures may affect food accessibility and may also trigger stress leading to a more frequent consumption of “comfort foods”. Results showed that during the lockdown, almost half of the respondents consumed sweets, 1/5 also had sweetened drinks in their diet, over 1/3 of the population consumed salty snacks, and a little over 60% consumed animal protein at least once a day. These results underline that in addition to the dietary transition seen the past decades in this region, the pandemic has further triggered unhealthy dietary choices that may further increase metabolic diseases, and the authors agree with Grabia and colleagues [2] and recommend dietary counseling to increase fruits, vegetables, and other foods close to the MD pattern. Moreover, the importance of dietary counseling on the lipid profile in healthy [6,7] and dyslipidemic individuals [3] was evident by intervention studies included in the present SI.

Metabolic Syndrome is one among many metabolic diseases with a dramatic rise in the past years. It has been closely linked with the transition to a more Westernized diet and is inversely associated with the MD. In this SI, this relationship was examined by Balakoudi and colleagues through a systematic review that assessed the impact of MD adherence on various parameters of the metabolic syndrome as defined by NCEP-ATP III criteria1. These parameters included waist circumference (WC), blood pressure, fasting blood glucose, HDL-c and TG, with a total of 41 observational studies included, summing to 74,058 adults. In total, 23 studies evaluated the level of MD adherence on WC with a significantly inverse pooled effect being found. HDL-c was assessed by 39 studies (*N* = 31,800 individuals) and the pooled effect showed increased levels with higher MD adherence, while TG were found significantly lower, in agreement with other studies included in the Special Issue [3,6,7]. 

It has to be mentioned at this point that to date there is no specific value that can be used to define the ideal level for high MD adherence, an aspect that needs to be further investigated, especially following results from the 6-month intervention study performed by Sotos-Prieto and colleagues, which resulted in favorable changes in lipid profile, even with a non-significant slight increase to the MD adherence [6]. 

The intervention study included in this SI addressed how it can ameliorate adherence to the MD diet and how this improvement, if any, affects specific metabolic biomarkers and other anthropometrics related to CVD in firefighters [6,7]. The authors concluded that the MD may promote beneficial changes in specific lipid species, including HDL-c, all LDL-c sizes, TG, total cholesterol and ApoA/ApoB ratio, due to the high content of mono- and polyunsaturated fatty acid found in this dietary pattern [6]. 

Collectively, this SI includes an array of studies from various countries that address directly or indirectly the effects of MD on health and additionally address the influence of the COVID-19 pandemic, the new challenge of the decade. It effectively summarizes not only effects of specific nutrients, such as sodium and saturated fats, but also of whole foods as well, on specific risk factors and biochemical indices, as well as to end-points of diseases. Finally, it highlights the importance of addressing the best way of defining high/low adherence to the MD and the need for population-specific interventions and potentially developing new and innovative uniform tools that will lead to an increase of adherence and preserve the MD pattern. 
